# Quad-Band 1 × 4 Linear MIMO Antenna for Millimeter-Wave, Wearable and Biomedical Telemetry Applications

**DOI:** 10.3390/s24144427

**Published:** 2024-07-09

**Authors:** Rakesh N. Tiwari, K. Geetha Malya, Girigari Nandini, P. Baby Nikhitha, Deepti Sharma, Prabhakar Singh, Pradeep Kumar

**Affiliations:** 1 Department of Electronics & Communication Engineering, Madanapalle Institute of Technology & Science, Madanapalle 517325, Andhra Pradesh, India; geethamalya1@gmail.com (K.G.M.); nandinigirigari@gmail.com (G.N.); nikhithapathe@gmail.com (P.B.N.); 2 Department of Electronics and Communication Engineering, G. L. Bajaj Institute of Technology and Management, Greater Noida 201306, Uttar Pradesh, India; deeptidec24@gmail.com; 3 Physics Division, School of Basic Sciences, Galgotias University, Greater Noida 203201, Uttar Pradesh, India; prabhakarsingh3@gmail.com; 4 Discipline of Electrical, Electronic and Computer Engineering, University of KwaZulu-Natal, Durban 4041, South Africa

**Keywords:** Linear MIMO antennas, quad band, mm-wave, diversity parameters, bending analysis, SAR, link budget, 5G and WBAN applications

## Abstract

In this paper, we present the design of a millimeter-wave 1 × 4 linear MIMO array antenna that operates across multiple resonance frequency bands: 26.28–27.36 GHz, 27.94–28.62 GHz, 32.33–33.08 GHz, and 37.59–39.47 GHz, for mm-wave wearable biomedical telemetry application. The antenna is printed on a flexible substrate with dimensions of 11.0 × 44.0 mm^2^. Each MIMO antenna element features a modified slot-loaded triangular patch, incorporating ‘cross’-shaped slots in the ground plane to improve impedance matching. The MIMO antenna demonstrates peak gains of 6.12, 8.06, 5.58, and 8.58 dBi at the four resonance frequencies, along with a total radiation efficiency exceeding 75%. The proposed antenna demonstrates excellent diversity metrics, with an ECC < 0.02, DG > 9.97 dB, and CCL below 0.31 bits/sec/Hz, indicating high performance for mm-wave applications. To verify its properties under flexible conditions, a bending analysis was conducted, showing stable S-parameter results with deformation radii of 40 mm (Rx) and 25 mm (Ry). SAR values for the MIMO antenna are calculated at 28.0/38.0 GHz. The average SAR values for 1 gm/10 gm of tissues at 28.0 GHz are found to be 0.0125/0.0079 W/Kg, whereas, at 38.0 GHz, average SAR values are 0.0189/0.0094 W/Kg, respectively. Additionally, to demonstrate the telemetry range of biomedical applications, a link budget analysis at both 28.0 GHz and 38.0 GHz frequencies indicated strong signal strength of 33.69 dB up to 70 m. The fabricated linear MIMO antenna effectively covers the mm-wave 5G spectrum and is suitable for wearable and biomedical applications due to its flexible characteristics.

## 1. Introduction

With the progress of the Internet of Things (IoT) and 5G communication, modern healthcare is advancing day by day. Therefore, modern communication devices and wearable biomedical telemetry systems need such frequency bands that transmit large data, ensuring the quality of audio and video signal [1]. At present, the multiple-input–multiple-output (MIMO) antennas in the millimeter frequency range are the suitable choice of researchers to transmit/receive the signals with high-speed data rate [2]. Since there are many advantages of the MIMO antenna over a single-element antenna, the MIMO designs are highly recommended for the high-speed 5G communication technologies [3]. However, there are certain constraints while designing the MIMO antenna such as the size of the antenna and the isolation between the radiating elements [4]. To address these issues, attempts were made in [5,6] to minimize the coupling effect between the MIMO elements in the proximity. In [7], a dual-band mm-wave MIMO antenna was designed for 28/38 GHz with good isolation, but the antenna has very narrow operating bands. The orthogonal elements in the MIMO design can largely improve the isolation as reported in [8], in which the composite radiating structures were used to operate at 28/38 GHz. In addition to that, a square slot was etched at the center of the ground plane to further improve the isolation > 33 dB at both the resonance frequencies. Along with the orthogonal arrangement of MIMO elements, the frequency selective surface (FSS) can be used to reduce the mutual coupling [9]. Due to the use of a 7 × 7 array of unit cells in FSS and printed at a suitable position in the MIMO design, the reported MIMO antenna exhibits the increased gain of 8.6 dBi and isolation > 26 at the operating frequency of 28 GHz. The implementation of a metamaterial structure in a coplanar MIMO structure is also one of the methods to improve the isolation. In [10], a split-ring resonator (SRR) with some modification was used between the two radiating elements. The dual-port MIMO antenna was operating at 28/38 GHz with isolation > 28 dB. A dual-band two-port mm-wave MIMO antenna was designed in [11] operating at 24/36 GHz. The isolation was improved by etching the complementary split-ring resonator (CSRR) structure in the ground plane. To improve the overall gain, an array of four radiating elements with corporate feed technique [12] was used to design the dual-port MIMO antenna covering the operating band of 37 GHz. This design demonstrates the enhanced gain of 12.8 dB with an overall antenna dimension of 20 × 40 × 0.254 mm^3^. In [13], multiple intersecting rings were used as MIMO elements to enhance the antenna gain up to 11 and 10.9 dBi at two resonant frequencies of 28 and 38 GHz, respectively. This 4-port MIMO design has linear arrangement of elements, and the total size of the antenna is 48 × 12 × 0.254 mm^3^. Four mm-wave array along with two sub-6 GHz radiating elements were utilized as a co-designed system [14]. Further, a PIN diode was used to address the issue of beam steering in mm-wave frequency range. This antenna design was working in the millimeter frequency range (27.5–28.35 GHz) and sub-6 GHz (0.79–0.96 GHz and 1.7–5 GHz) applicable for 5G smartphones. Moreover, the PIN diode and shorting pins were used along with an H-shaped slot in the ground plane of the MIMO antenna to achieve the dual-frequency band [15]. In the upcoming advancement in wireless technology, the wearable MIMO antennas are widely used in the field of robotics, IoT, biotechnologies, medical sectors, and many more [16,17,18]. A semi-flexible 2-port MIMO antenna working in the 27 GHz and 29 GHz frequency bands was proposed in [19]. This antenna had a size of 36 mm × 22.5 mm and was proposed for biomedical applications. Recently, a 4-port mm-wave antenna of size 18 mm × 8.5 mm for wearable application was proposed in [20]; this antenna was working in the dual-frequency bands 28 GHz and 38 GHz.

Therefore, it can be found that the multiple-band MIMO antenna in the millimeter frequency range along with the flexible configuration is a very promising factor to facilitate the high-speed wireless communication in wearable electronics.

In this paper, we have designed, fabricated, and analyzed a quad-port flexible MIMO antenna operating in the mm-wave frequency range. The proposed antenna has a novel configuration with perturbed triangular-shaped radiating elements and two ‘cross’-shaped slots in the ground plane. A horizontal arrangement of 1 × 4 MIMO elements exhibits multiband characteristics. The significant diversity parameters such as envelope correlation coefficient (ECC), diversity gain (DG), mean effective gain (MEG), total active reflection coefficient (TARC), and channel capacity loss (CCL) of the MIMO antenna are calculated using CST Microwave Studio, and a prototype antenna is fabricated to compare the measured results. The manuscript is composed of nine sections, starting with the introduction followed by antenna design configuration in Section 2. In Section 3, S-parameters analysis and current distribution are performed, and Section 4 describes the realized gain, efficiency, and radiation pattern measurement. Diversity parameters are calculated in Section 5, and the bending analysis of the MIMO design is presented in Section 6. Furthermore, Section 7 presents the SAR calculation details to ensure the antenna’s suitability for on-body wearable applications. Section 8 discusses the link budget analysis for verifying high data rate telemetry. The manuscript concludes with the findings presented in Section 9.

## 2. Antenna Design Evolution and Analysis

**Figure 1:**
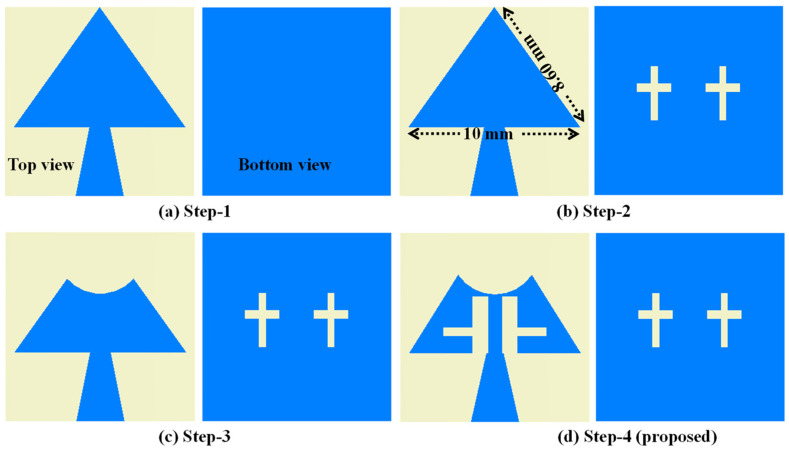
Antenna design evolution steps.

**Figure 2:**
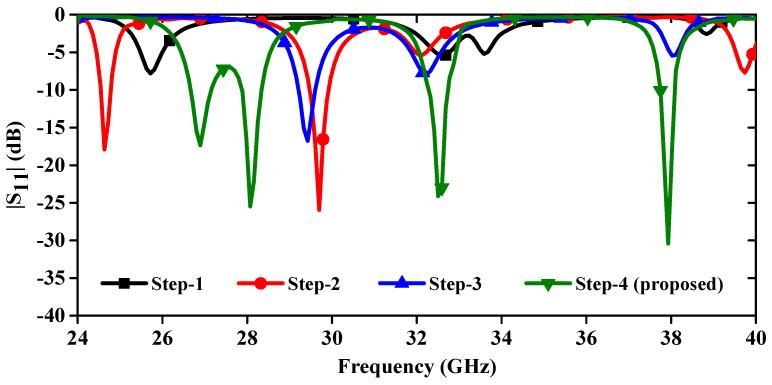
|S_11_| curves for various antenna designs.

The proposed mm-wave MIMO antenna optimization is successfully achieved in four different steps (Figure 1) and is evidenced by the corresponding |S_11_|-parameters in Figure 2. In Figure 1a, a triangular patch measuring 10 × 8.60 mm^2^ (top view) is connected to the tapered microstrip feed line. A full rectangular ground plane is used on the bottom of the substrate. Initially, this antenna’s first resonance was at 11.8 GHz; other resonances were in mm-wave (24–40 GHz). This antenna was designed using the equation given in [21]. The theoretical resonating frequency at the 10 mm side length is 11.8 GHz, which is close to the simulated value of 12.7 GHz. This discrepancy might be due to tapered feed, which improved impedance matching in the mm-wave frequency bands because our primary objective was to design an antenna for mm-wave applications.

In Step 1, the |S_11_|-parameters reveal multiple resonances, indicating the presence of desired frequency responses. However, the impedance matching is suboptimal. In step 2, inserting two cross-shaped slots into the ground plane, as depicted in Figure 1b, causes the resonance frequency to shift lower. This shift is due to the increased current path length. Additionally, two resonant dips, each below −10 dB, are observed. The cross-shaped slot has its horizontal and vertical dimensions 2.0 × 0.5 mm^2^ and 3.15 × 0.4 mm^2^, respectively. Furthermore, in step 3, introducing a circular notch with a radius of 2.58 mm into the top of the radiating triangular patch (Figure 1c) shifts the resonance points to the right. This shift is due to the reduction in patch dimensions, which also disrupts impedance matching. In the final step, two vertical slots measuring 3.3 × 0.9 mm^2^ and a horizontal slot measuring 1.7 × 0.5 mm^2^ are integrated into the radiating patch (as shown in Figure 1d). This adjustment enables the antenna to operate within the desired frequency bands. Specifically, the antenna’s |S_11_|-parameters are determined to fall within the operating frequency ranges of 26.68–27.12 GHz, 27.85–28.33 GHz, 32.28–32.71 GHz, and 37.71–38.10 GHz, as depicted in Figure 2. 

**Figure 3:**
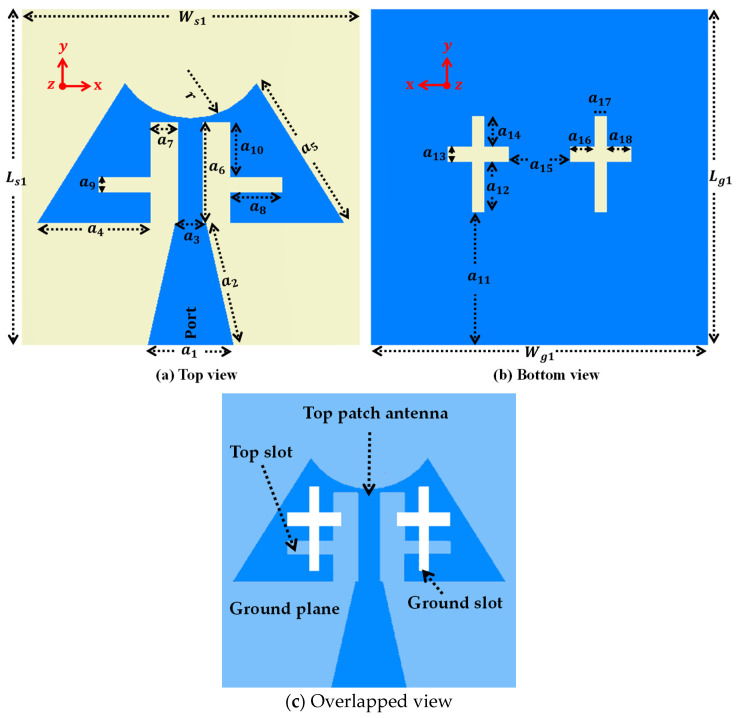
Single antenna design.

This frequency bandwidth is a result of extending the current path within the patch. The single-element antenna’s overall dimensions measure 11 × 11 × 0.25 mm^3^, with a flexible substrate Rogers 3003 (thickness 0.25 mm) material having relative permittivity ($\varepsilon_{r}$) of 3.0 and a loss tangent (tanδ) of 0.001, as depicted in Figure 3. The optimized parameters are given in Table 1.

## 3. 1 × 4 mm-Wave Linear MIMO Array Antenna Design and Measurement

**Figure 4:**
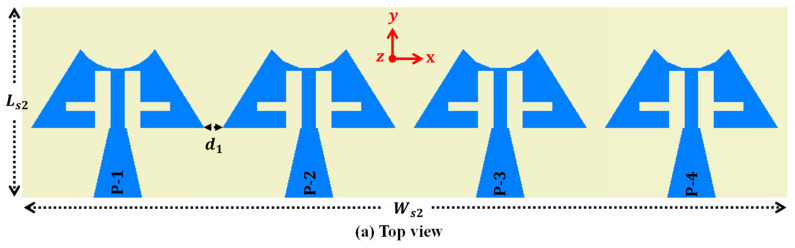

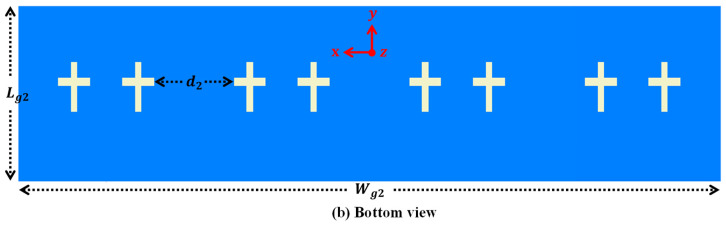
1 × 4 MIMO array antenna.

**Figure 5:**
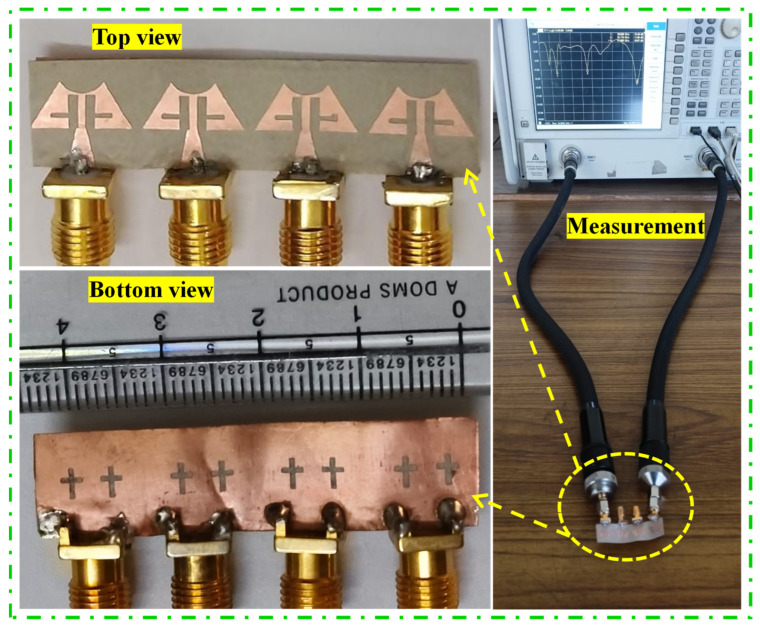
Prototype antenna and S-parameter measurement setup.

**Figure 6:**
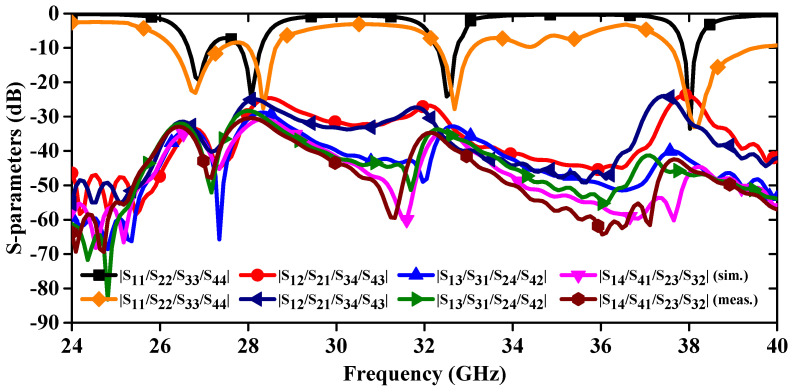
Simulated and measured S-parameters of 1 × 4 linear MIMO array antenna.

The antenna design is now transformed from a single optimized element to a 1 × 4 linear MIMO array antenna, maintaining a compact size with dimensions of 11.0 × 44.0 mm^2^ (Figure 4a). Each element within the array is spaced precisely 1.0 mm apart, ensuring optimal performance. Additionally, the ground plane features a series of cross-shaped slots, strategically positioned with a 5.0 mm separation between each adjacent pair (Figure 4b). Figure 5 illustrates the prototype of the proposed MIMO antenna, and S-parameter measurements are conducted to confirm the accuracy of the simulated results. In Figure 6, a comparison is drawn between the S-parameter results obtained from simulation and measurement. The simulated and measured resonances are found at 26.89 GHz and 26.82 GHz, 28.07 GHz and 28.34 GHz, 32.51 GHz and 32.68 GHz, and 38.02 GHz and 38.11 GHz, respectively. The slight variance observed between the simulated and measured resonance frequencies may be due to inconsistencies in soldering error and fabrication processes. Furthermore, the proposed frequency bands are part of the mm-wave spectrum, which are increasingly being utilized for various advanced communication applications [22]. The first operating band covering 26.28–27.36 GHz is part of the 5G NR (New Radio) frequency range 2 (FR2). It is used for high-speed data transmission, providing enhanced mobile broadband and supporting ultra-reliable low-latency communications. The second band occupies a 27.94–28.62 GHz frequency range and supports applications like enhanced mobile broadband (eMBB) and fixed wireless access (FWA). FWA provides high-speed internet connectivity to homes and businesses, especially in areas where fiber or cable deployment is not feasible. Further, the third band (32.33–33.08 GHz) is part of the Ka-band, which is widely used for satellite communications, including both commercial and military applications. It supports high-capacity data transmission for internet, television, and other communication services. The last operating band covering 37.59–39.47 GHz is also part of the 5G NR FR2 and is used for very high-speed wireless communication such as augmented reality (AR), virtual reality (VR), and industrial automation.

The proposed antenna exhibits excellent isolation values, surpassing 24 dB across all operational frequency bands. This outstanding performance makes it well-suited for employment in both mm-wave applications and mm-wave wearable biomedical applications.

**Figure 7:**
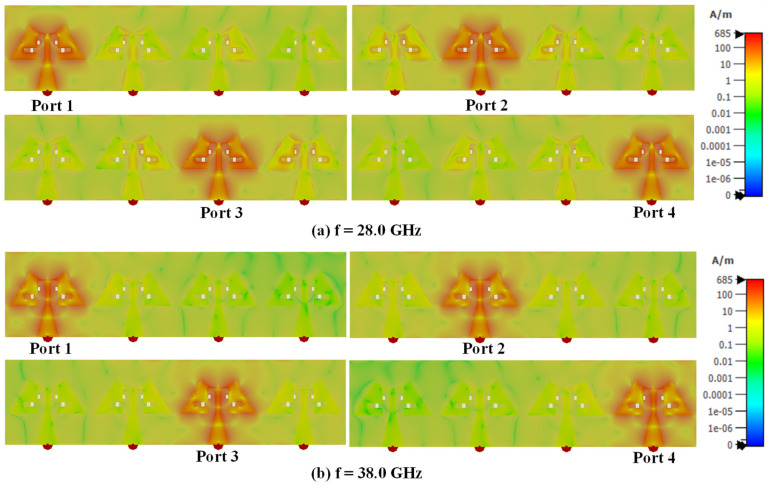
Surface current distribution at 28.0 and 38.0 GHz.

The surface current density for the MIMO antenna is evaluated at 28.0 GHz and 38.0 GHz (Figure. 7). The current density around the periphery including the slots in the patch is higher (Figure 7a), which elongates the effective length of the patch leading to resonate the antenna at 28 GHz. At 38 GHz, the current distribution shows null points (Figure 7b), indicating the higher mode generated in the modified triangular patch. From the current distribution graph at 28/38 GHz, it is evident that there is minimal coupling between adjacent ports, even in the absence of decoupling elements.

## 4. 1 × 4 mm-Wave Linear MIMO Array Antenna Gain, Radiation Efficiency and Radiation Pattern

**Figure 8:**
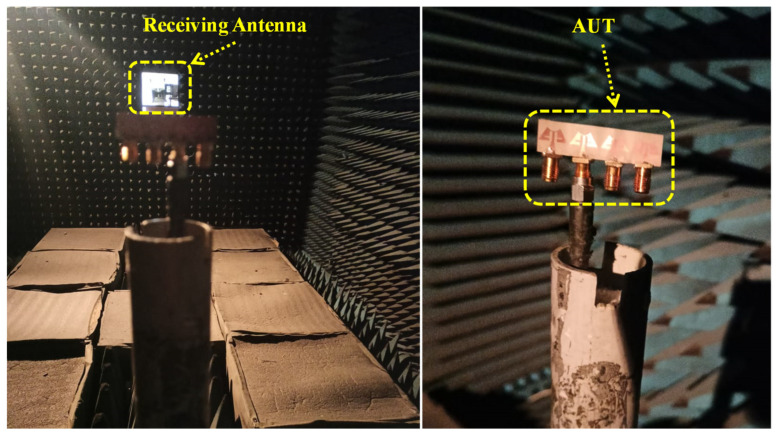
Experimental setup of gain measurement.

**Figure 9:**
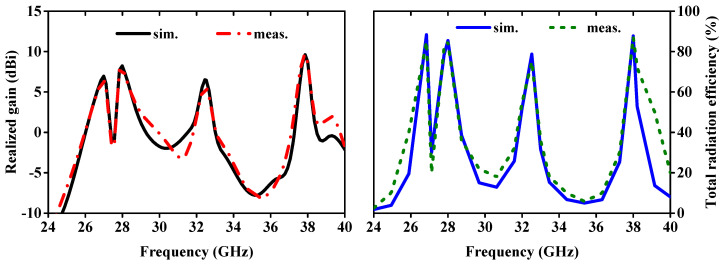
Simulated and measured realized gains and total radiation efficiencies.

The antenna gain measurement setup is shown in Figure 8. Figure 9 (left side) presents the simulated and measured realized gain. The measured peak gains for the antenna at four different frequency bands are 6.12 dBi (26.28–27.36 GHz), 8.06 dBi (27.94–28.62 GHz), 5.58 dBi (32.33–33.08 GHz), and 8.58 dBi (37.59–39.47 GHz). In comparison, the simulated peak values are 6.42 dBi (26.68–27.12 GHz), 8.17 dBi (27.85–28.33 GHz), 6.46 dBi (32.28–32.71 GHz), and 8.72 dBi (37.71–38.10 GHz). The total radiation efficiency (TRE) of the proposed quad-port MIMO array antenna, both simulated and measured, is depicted in Figure 9 (right side). At resonance points, the peak values of TRE are observed to be 88.36% and 85.0%, 85.43% and 84.67%, 78.75% and 75.0%, and 87.75% and 87.0% for the simulated and measured data, respectively. The radiation efficiencies at two edges of each operating bands of 26.28–27.36 GHz, 27.94–28.62 GHz, 32.33–33.08 GHz, and 37.59–39.47 GHz are found to be 76.24 and 85.20%, 80.77 and 72.14%, 64.81 and 60.24%, and 65.86 and 65.33%, respectively.

**Figure 10:**
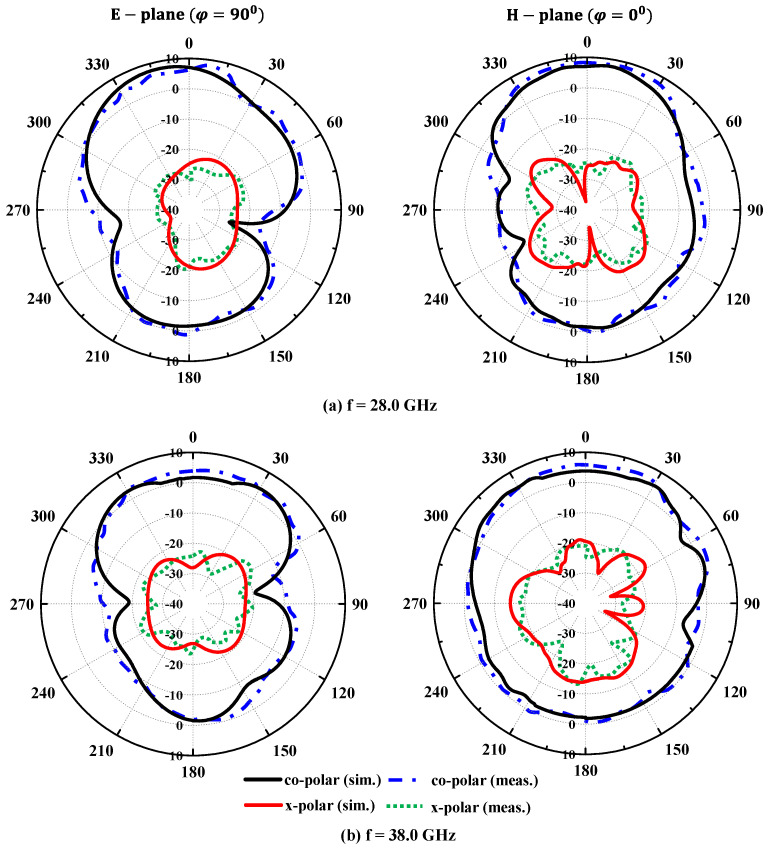
Comparison of radiation patterns at (**a**) 28.0 GHz and (**b**) 38.0 GHz.

The far-field patterns of the MIMO antenna are measured in the anechoic chamber (dimension 12 × 25 square feet). The horn antenna (with maximum gain of 20 dBi) is used as the receiving end, and the 1 × 4 MIMO antenna is placed on a rotating platform under test (Figure 8). Here, we are measuring the radiation properties of the MIMO antenna specifically at 28.0/38.0 GHz frequencies, which are widely employed in millimeter-wave applications. The E-plane pattern at an azimuthal angle of 90^0^ is shown in Figure 10. The simulated and measured co- and cross-polarization patterns exhibit excellent agreement. At an elevation angle of 0^0^, there is a substantial difference of over −20 dB between co- and cross-polarization levels across both operating frequencies. Similar results are found for H-plane patterns at an azimuthal angle of 0^0^. Hence, based on this analysis, it is evident that the proposed MIMO design exhibits outstanding radiation patterns suitable for wearable applications.

## 5. Diversity Parameters of mm-Wave 1 × 4 Linear MIMO Array Antenna

**Figure 11:**
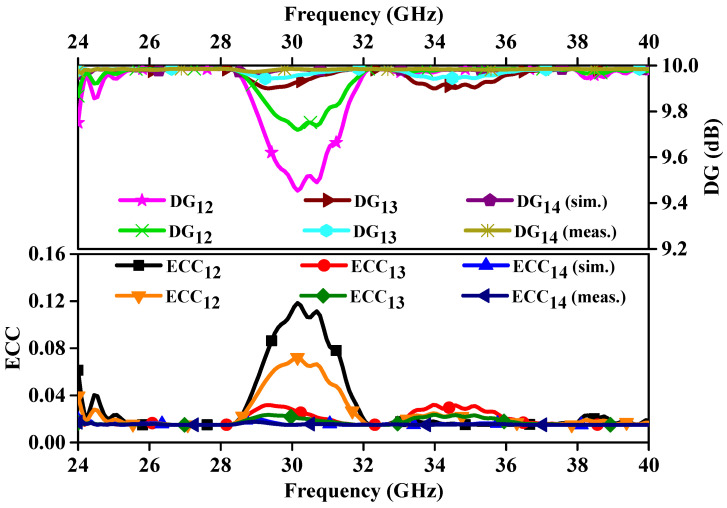
Simulated and measured ECC and DG.

In this section, the key diversity parameters of the MIMO antenna are calculated and compared with the measured results. In Figure 11, we assess the ECC and DG across all operational bands. Here, it should be noted that the ECC value is calculated using 3-D far-field radiation patterns [19]. From Figure 11, we can see that the DG between different ports lies above 9.97 dB throughout the applicable band. Additionally, the ECC is also less than 0.02 across all the reasoning bands. The DG and ECC values are under the limit of acceptable values in MIMO systems.

**Figure 12:**
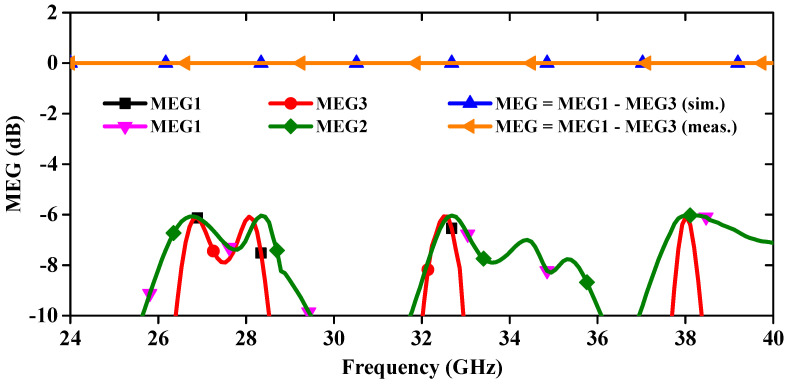
Simulated and measured curves of MEG.

MEG is a crucial parameter for assessing the potential application of MIMO antennas in 5G and wearable communication systems. It is defined as the ratio of the mean received power to the average incident power for a linear MIMO array antenna, compared to that of an isotropic antenna. The MEG is determined using far-field radiation patterns, as shown in Figure 12. Both simulated and measured MEG values are ≤−6.05 dB across all relevant frequency bands, which is below the acceptable limit of −3 dB [23]. 

**Figure 13:**
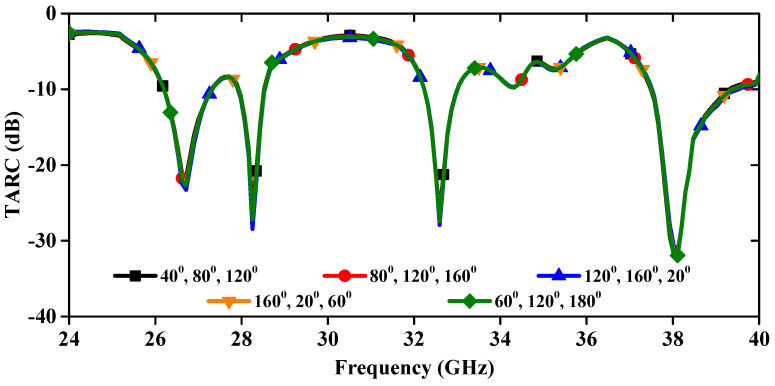
Calculated TARC of linear MIMO array antenna.

The TARC serves as a crucial metric within MIMO antenna systems, signifying the balance between incident and reflected signals. Mathematically, TARC is determined by [24], as depicted in Figure 13. This figure reveals that with a consistent increment in the phase angle of the input signal, TARC is stable without deviation.

**Figure 14:**
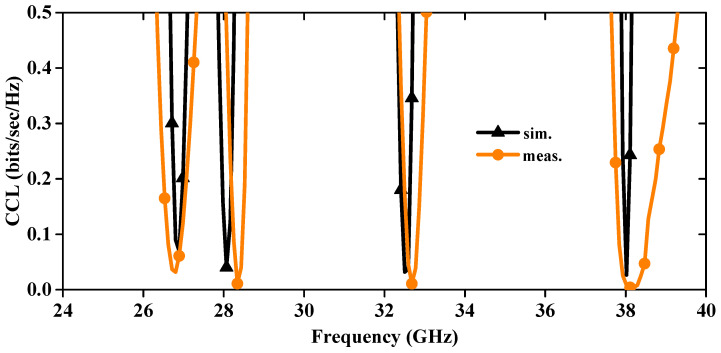
Simulated and measured CCL of MIMO antenna.

The CCL is also another significant parameter of MIMO antenna, and it measures the signal alteration between the transmitter and receiver end. The CCL value is mathematically expressed in [25], and the simulated and measured graph is illustrated in Figure 14. The simulated and measured CCL values across four bands are <0.29 and 0.31, 0.32 and 0.34, 0.28 and 0.31, and 0.24 and 0.27 bits/sec/Hz, respectively. Notably, these values consistently remain below 0.4 bits/sec/Hz [26], aligning with the established threshold value for MIMO antenna performance.

## 6. Bending Analysis of mm-Wave Linear MIMO Array Antenna

**Figure 15:**
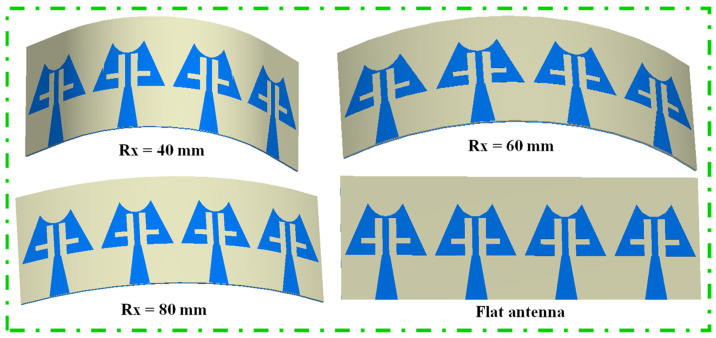
Bending configuration of MIMO array antenna in x-direction.

**Figure 16:**
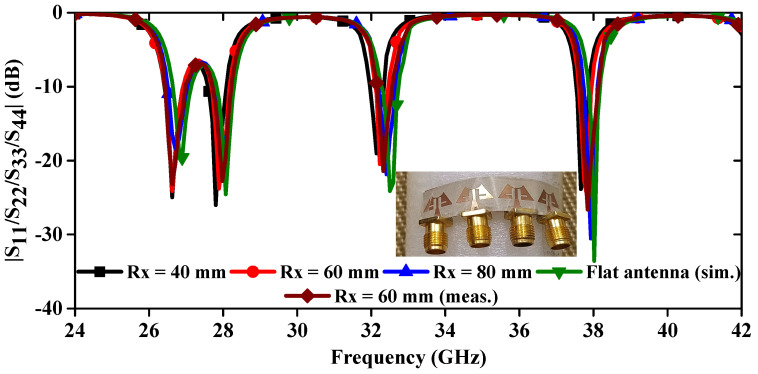
Simulated and measured |S_11_/S_22_/S_33_/S_44_| curves at different radii along x-directions.

**Figure 17:**
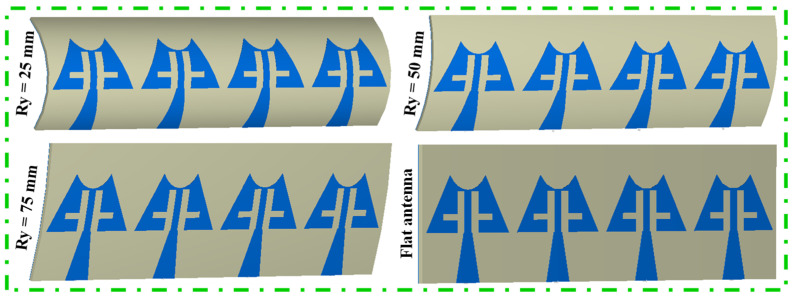
Bending configuration of antenna along y-direction.

**Figure 18:**
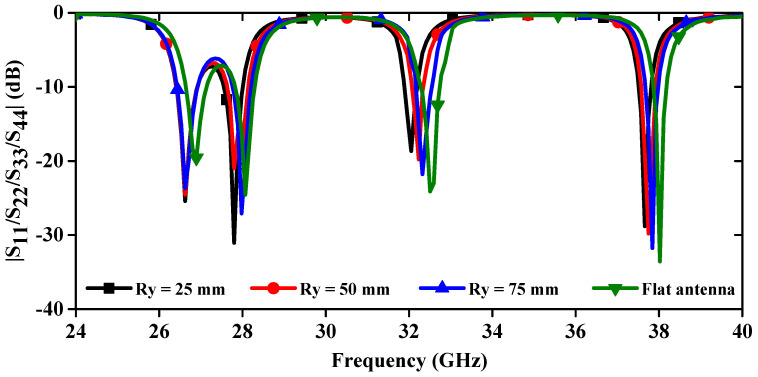
Simulated |S_11_/S_22_/S_33_/S_44_| curves at different radii along y-directions.

**Figure 19:**
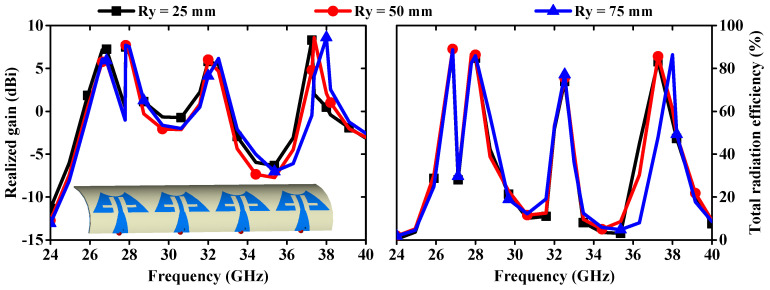
Realized gains and total radiation efficiencies at different bending conditions.

The deformation of the antenna structure can alter its properties. To examine the S-parameter behavior of the MIMO antenna, a bending analysis along the x-direction was conducted, as shown in Figure 15. Additionally, Figure 16 presents the |S_11_/S_22_/S_33_/S_44_| curves plotted for different bending radii along the x-axis. Figure 16 shows that the resonance dips are slightly altered up to Rx = 40 mm, and the measured bending results match the simulated results. When bending in the y-direction is analyzed as depicted in Figure 17, it is observed that again a small change in resonance frequencies is observed even at the worst case when Ry = 25 mm (Figure 18). Moreover, the realized gains and radiation efficiencies are calculated at Ry = 25, 50, and 75 mm, and the corresponding results are depicted in Figure 19. Slight variations in peak gains and efficiencies are observed due to the change in the overall impedance of the MIMO antenna, which result from alterations in current density in the deformed structure of the MIMO design. This analysis indicates that the proposed mm-wave MIMO array antenna is suitable for 5G, wearable, and medical applications.

## 7. Specific Absorption Ratio (SAR) Analysis

**Figure 20:**
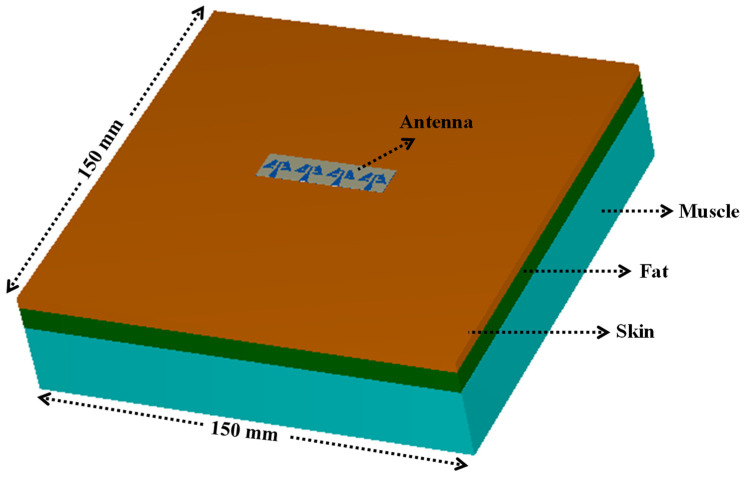
Proposed 1 × 4 MIMO antenna on cuboid phantom for SAR calculation.

**Figure 21:**
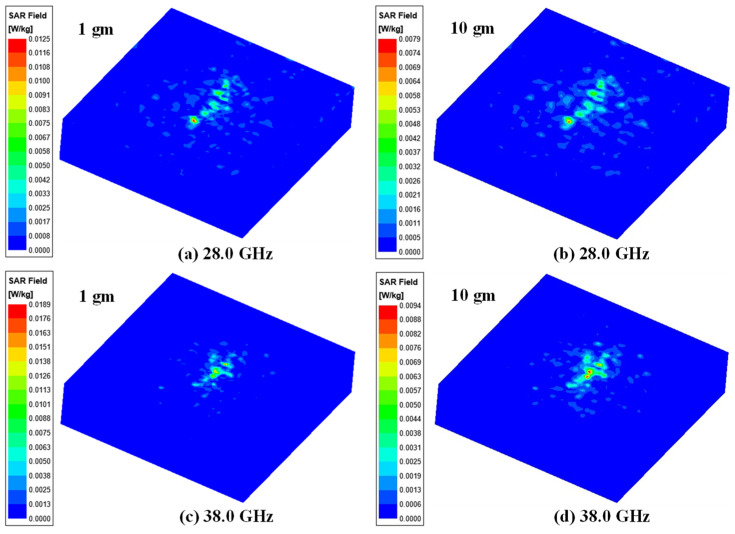
SAR values of 1 × 4 MIMO array antenna.

SAR analysis is mandatory when a MIMO antenna is placed on a human body as the side or back lobes’ electromagnetic radiation of the antenna may get absorbed in the body. The constant excessive exposure to EM wave radiation might affect the human tissues and causes health issues [27,28]. Therefore, to ensure safety from such EM wave-radiating devices, IEEE C95.1-2019 and the International Commission on Non-Ionizing Radiation Protection (ICNIRP) [29] have standardized the peak average SAR < 1.6 and 2.0 W/kg over 1 and 10 g of tissues, respectively. To evaluate the SAR values for the proposed MIMO array design, a cuboid multilayer phantom of 150 × 150 mm^2^ with muscle, fat, and skin of thickness 25, 8, and 4 mm [30,31], respectively, is used in CST Microwave Studio simulator, as shown in Figure 20. The electrical properties of three layers of tissue (muscle, fat, and skin) are presented in Table 2. The average SAR (ASAR) at 1 and 10 gm (in W/kg) tissues are estimated (Figure 21), and the corresponding values are depicted in Table 3 at an input power of 2.5 mW. The maximum ASAR and the acceptable power at 28.0 GHz for 1/10 gm values are 0.0125/0.0079 W/Kg and 320.00/632.91 mW, respectively, whereas, at 38.0 GHz, the maximum ASAR and corresponding power values are 0.0189/0.0094 W/Kg and 211.64/531.91 mW, respectively. From Table 3, it can be observed that the calculated ASAR is less than the acceptable limit, and hence, the 1 × 4 linear MIMO array antenna is appropriate for mm-wave and wireless body area network (WBAN) applications.

## 8. Link Budget Study

The proposed mm-wave 1 × 4 linear MIMO array antenna is versatile and can be employed in a range of applications, including 5G networks, biomedical wearable communication technologies, and other WBANs. We conducted a link budget analysis to determine the effective mm-wave range over which the proposed antenna can reliably connect with another device. The communication link is influenced by various parameters, including S-parameters, path loss, absorption, and mismatch losses. Therefore, it is essential to study the link margin (LM) in order to estimate the actual communication range of the designed antenna. The parameters considered for the link budget are listed in Table 4 [19,33].

**Figure 22:**
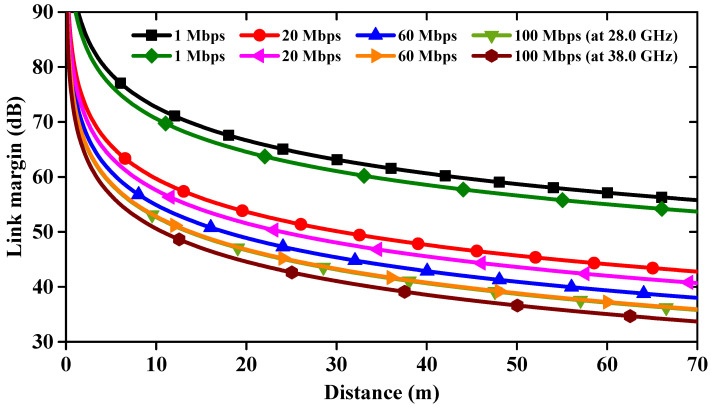
Link margin versus distance for the proposed MIMO antenna.

Based on Table 4, the LM was calculated and is depicted in Figure 22. From this figure, it is evident that as the distance increases, the LM decreases. This MIMO antenna design demonstrates successful communication over a distance of 70 m at both 28 GHz and 38 GHz frequencies. This is achieved with a very low transmitter input power, meeting the maximum Effective Isotropic Radiated Power (EIRP) regulations for mm-wave and WBANs. The system also claims an LM exceeding 33.69 dB at both frequencies, indicating a strong signal strength and a significant buffer against signal degradation. The proposed design demonstrates superior results in SAR analysis, MEG, CCL, and link budget analysis compared to findings in the existing literature (Table 5).

Table 5 shows the comparison of the proposed antenna with the various existing mm-wave antennas. The proposed antenna has four ports, which is better than the antennas with fewer ports, as more ports provide more available channels for communication. The presented 4-port antenna is compact in size when compared with the 4-port antennas presented in [1,8,9,13,20]. The 4-port antenna presented in [16] has less isolation of 18.89 dB as compared to the isolation of 24 dB by the proposed 4-port antenna. Therefore, it can be concluded that the proposed antenna has a simple, novel, and compact configuration with 4-port operation and high isolation (>24 dB) between the antenna elements. All these qualities make the proposed antenna suitable for mm-wave applications.

## 9. Conclusions

In conclusion, we have designed a mm-wave 1 × 4 linear MIMO array antenna featuring a slotted triangular radiating patch and a defected ground plane. This MIMO antenna effectively covers multiple resonance frequencies (26.82, 28.34, 32.68, and 38.11 GHz) through precise positioning of the slots in the radiating elements and careful tuning of the dimensions in the ground plane. Additionally, the antenna achieves isolation of over 24 dB between adjacent ports without the need for additional decoupling techniques. The analysis of ECC, DG, MEG, TARC, CCL, and bending characteristics and its ultra-thin, flexible design confirms the antenna’s suitability for high data rate (100 Mbps up to a distance of 70 m at 28.0/38.0 GHz) 5G and wearable biomedical communication technologies. The SAR evaluation on a multilayer phantom reveals values well below critical limits for both 1 gm and 10 gm of tissue, ensuring safe use for wearable biomedical applications.

## Figures and Tables

**Table 1:** Design parameters of the antenna.

Parameter	$L_{s1}$	$W_{s1}$	$a_{1}$	$a_{2}$	$a_{3}$	$a_{4}$	$a_{5}$	$a_{6}$	$a_{7}$	$a_{8}$
Value (mm)	11.0	11.0	2.8	4.1	1.0	3.7	5.39	3.3	0.9	1.7
Parameter	$a_{9}$	$a_{10}$	r	$L_{g1}$	$W_{g1}$	$a_{11}$	$a_{12}$	$a_{13}$	$a_{14}$	$a_{15}$
Value (mm)	0.5	1.8	2.58	11.0	11.0	4.35	1.65	0.5	1.0	2.0
Parameter	$a_{16}$	$a_{17}$	$a_{18}$	$L_{s2}$	$W_{s2}$	$d_{1}$	$L_{g2}$	$W_{g2}$	$d_{2}$	*-*
Value (mm)	0.8	0.4	0.8	11.0	44.0	1.0	11.0	44.0	5.0	*-*

**Table 2:** Electrical properties of body tissues [32].

	28.0 GHz		38.0 GHz	
	$\mathrm{Permittivity}\;(\varepsilon_{r})$	Conductivity (σ) in S/m	$\mathrm{Permittivity}\;(\varepsilon_{r})$	Conductivity (σ) in S/m
**Muscle**	24.4	33.6	19.1	41.8
**Fat**	6.09	5.04	5.33	6.36
**Skin**	16.6	25.8	12.3	31.0

**Table 3:** Maximum average SAR of the proposed 1 × 4 MIMO array antenna.

Frequency (GHz)	SAR (W/Kg) (Input Power = 2.5 mW)		Maximum Acceptable Power (mW)		Maximum Acceptable Power (dBm)	
	1 gm	10 gm	1 gm	10 gm	1 gm	10 gm
28.0	0.0125	0.0079	320.00	632.91	25.05	28.01
38.0	0.0189	0.0094	211.64	531.91	23.26	27.26

**Table 4:** Link Budget.

**Transmitter**		
	Frequency (GHz)	28.0/38.0
$G_{t}$	Antenna gain (dBi)	8.17/8.73
$P_{t}$	Transmitted power (dBm)	10
	EIRP (dBm)	18.17/18.73
**Receiver**		
$G_{r}$	Receiver antenna gain (dBi)	4.1
$T_{o}$	Ambient temperature (K)	293
k	Boltzmann constant	1.38 × 10^−23^
$N_{0}$	Noise power density (dB/Hz)	−203.9
**Signal quality**		
$B_{r}$	Bit rate (Mb/s)	1, 20, 60, 100
	Bit error rate	1 × 10^−5^
$E_{b}/N_{0}$	Ideal PSK (dB)	9.6
$G_{c}$	Coding gain (dB)	0
$G_{d}$	Fixing deterioration (dB)	2.5

**Table 5:** Comparison of the proposed antenna with latest reported antennas.

Ref./ Year	No. of Ports	Volumetric Size (mm^3^)	Frequency Band (GHz)	Isolation (dB)	Max. Peak Gain (dBi)	ECC	MEG (dB)	CCL (Bits/s/Hz)	Bending Analysis	SAR (1/10 g)	Link Budget
[1] (2023)	4	25 × 25 × 0.787 (491.88)	25.28–28.02	>23.2	8.72	<0.0015	NR	NR	NR	NR	NR
[5] (2023)	2	30 × 15 × 0.203 (91.35)	28/38 (center frequency)	>32.3	6.9	<10^−4^	NR	<0.4	NR	NR	NR
[6] (2023)	2	26 × 14 × 0.762 (277.37)	27.1–28.8/ 35.2–38.9	>34.6	6.5	0.2 × 10^−4^	NR	NR	NR	NR	NR
[8] (2023)	4	26 × 26 × 0.25 (169.00)	27.7–28.3/ 37.7–38.3	>33	8.1	<0.0005	NR	<0.0064	NR	NR	NR
[9] (2023)	4	38 × 36 × 0.8 (1094.40)	27.2–28.85	>26.31	7.2	<0.002	NR	NR	NR	NR	NR
[10] (2024)	2	18 × 9.2 × 0.787 (130.33)	28/38 (center frequency)	>30	7.8	<0.0001	NR	<0.05	NR	NR	NR
[11] (2024)	2	8 × 15 × 0.8 (96.0)	23.5–25.5/ 34.2–36.4	>15	8.5	<0.005	Value not given	<0.315	NR	NR	NR
[13] (2023)	4	48 × 12 × 0.254 (146.30)	23–33/ 37.75–41	>20	5.7	<0.00015	<3.13	NR	NR	NR	NR
[15] (2020)	1	25 × 15 × 0.762 (285.75)	27.7–28.7/ 36.8–40.2	NA	9.0	NA	NA	NA	NA	NA	NA
[16] (2024)	4	16 × 12 × 0.25 (48.0)	27.43–28.41/ 37.67–38.56/ 40.11–41.17	>18.89	7.59	<0.025	NR	NR	Yes	NR	NR
[18] (2022)	1	29.09 × 11.42 × 0.508 (168.76)	27–29	NA	11.0	NA	NA	NA	NA	1.07	NA
[19] (2023)	2	36 × 22.5 × 0.508 (411.48)	24–31	>25	6.1	<0.003	−6	<0.26	Yes	1.64/2.18	>10
[20] (2024)	4	18 × 8.5 × 0.25 (38.25)	27.76–28.48/37.69–38.19	>20	7.73	<0.03	−6	<0.15	Yes	0.11/0.08 and 0.05/0.04	>33
Prop. work	4	44 × 11 × 0.25 (121.0)	26.28–27.36/ 27.94–28.62/ 32.33–33.08/ 37.59–39.47	≥24	8.58	<0.02	≤−6.05	<0.31	Yes	0.0125/ 0.0079 and 0.0189/ 0.0094	>33.69 (up to 70 m)

Note: NA—Not Applicable, NR—Not Reported.

## Data Availability

Complete data is available in the research paper.
